# Supplementary material for: Novel ABC Transporter Associated with Fluconazole Resistance in Aging of Cryptococcus neoformans
Source: J Fungi (Basel). 2022 Jun 28;8(7):677. doi: 10.3390/jof8070677 (PMC9320417; doi:10.3390/jof8070677)
Supplement: Supplementary file 1 [file jof-08-00677-s001.zip › jof-1789346-supplementary.pdf]

Table S1. List of Primers

AFR3 + p*GAL1* F	CCTCTATACTTTAACGTCAAGGAGAAAAAAATGTCTGCCATCGCCATCGA
AFR3 + *CYC1* tt R	TGAATGTAAGCGTGACATAACTAATTACATGATTTACTTGCGCAACGTGAGAG
AFR3 F	ATGTCTGCCATCGCCATCGA
AFR3 R	TTACTTGCGCAACGTGAGAG
qPCR *AFR3* F	GCCCATAGCACTAGGAGTCG
qPCR *AFR3* R	ATGTCATCCTGCGAATAGCC
Sc *ACT1* F	ATGGATTCTGAGGTTGCTGC
Sc *ACT1* R	GGTGTCTTGGTCTACCGACG
NBD + *pGAL1* F	CCTCTATACTTTAACGTCAAGGAGAAAAAAATGCTTTGATCAAATCTTCCTTC TGGC
GFP F	CCTCTATACTTTAACGTCAAGGAGAAAAAAAAGCTTTTTAATTAACAGTAAA GGAGA
GFP R	TGAATGTAAGCGTGACATAACTAATTACATGATGGCGCGCCCTATTGTATAGT TCATCC
AFR3 GFP R	AACAAGAATTGGGACAACTCCAGTGAAAAGTTCTTCTCCTTTACTCTTGCGCA ACGTGAGAGTGAGCCA
Cn *ACT1* F	CCCACACTGTCCCCATTTAC
Cn *ACT1* R	AACCACGCTCCATGAGAATC
qPCR *AFR1* F	CTTTCCGAGCTGGTGAACTC
qPCR *AFR1* R	CACCTTCGATCACACCAATG
qPCR *AFR2* F	GGTTCCGACTACATGGCTGT
qPCR *AFR2* R	GAGTTCACCAGCTCGGAAAG
qPCR *MDR1* F	CTCTTGATCACATCGCGAAA
qPCR *MDR1* R	ACCGACAATCTTGCTCTGCT

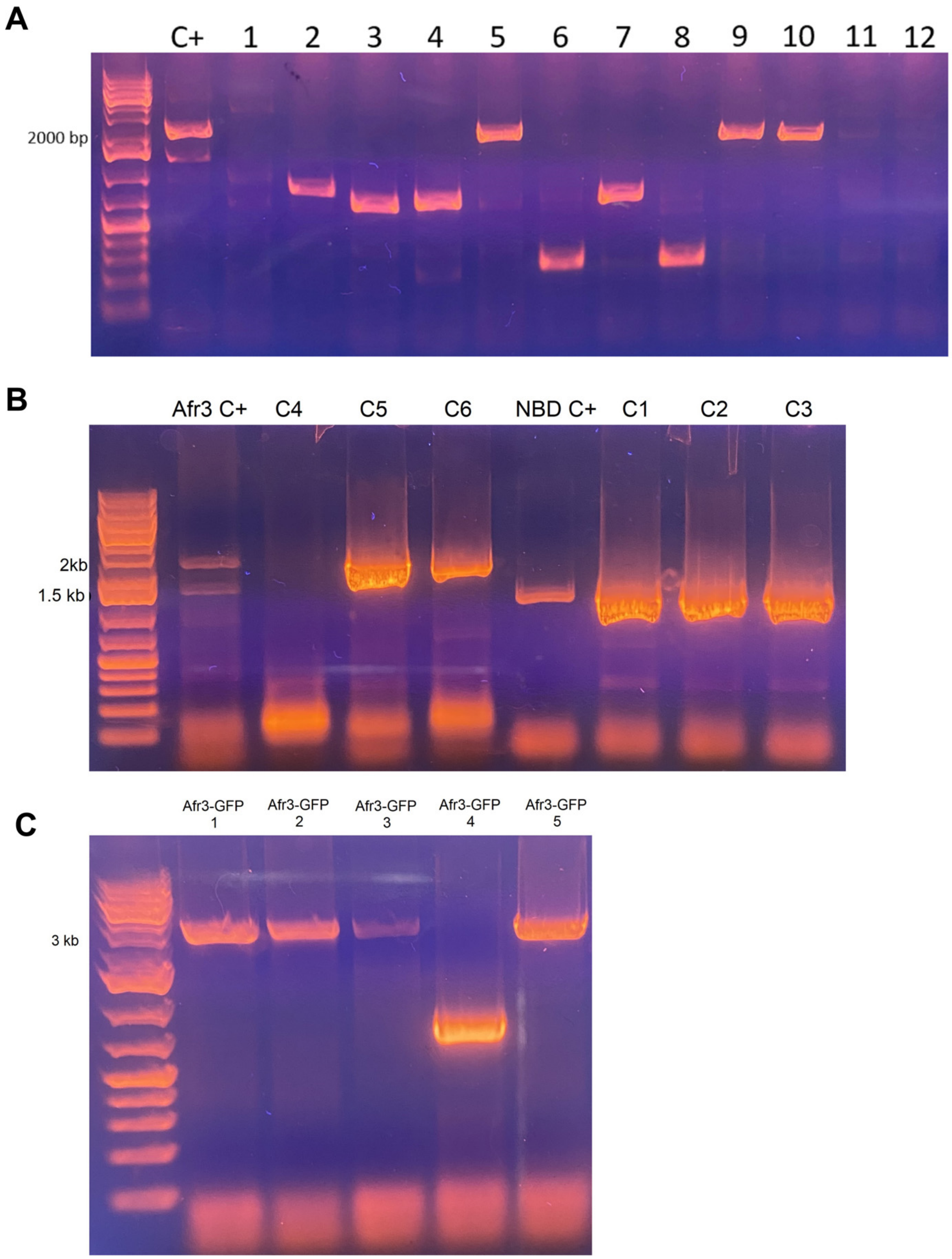
A
C+
1
2
3
4
5
6
7
8
9
10
11
12
2000 bp
B
Afr3 C+
C4
C5
C6
NBD C+
C1
C2
C3
2kb
1.5 kb
C
Afr3-GFP 1
Afr3-GFP 2
Afr3-GFP 3
Afr3-GFP 4
Afr3-GFP 5
3 kb

Figure S1. Confirmation of *Saccharomyces cerevisiae* Transformants. (**A**) Plasmids were extracted from ADΔ cells and PCR was performed for the Afr3 cassette. PCR samples were run in a 0.8 % agarose gel and positive colonies were identified by the correct size of amplification (2 kb); (**B**) Plasmids were extracted from ADΔ cells and PCR was performed for the Afr3 cassette and the Afr3-NBD cassette. PCR samples were run in a 0.8 % agarose gel and positive colonies were identified by the correct size of amplification (Afr3 = 2 kb, Afr3-NBD = 1.5 kb); (**C**) Plasmids were extracted from ADΔ cells and PCR was performed for the Afr3-GFP cassette. PCR samples were run in a 0.8 % agarose gel and positive colonies were identified by the correct size of amplification (3 kb).

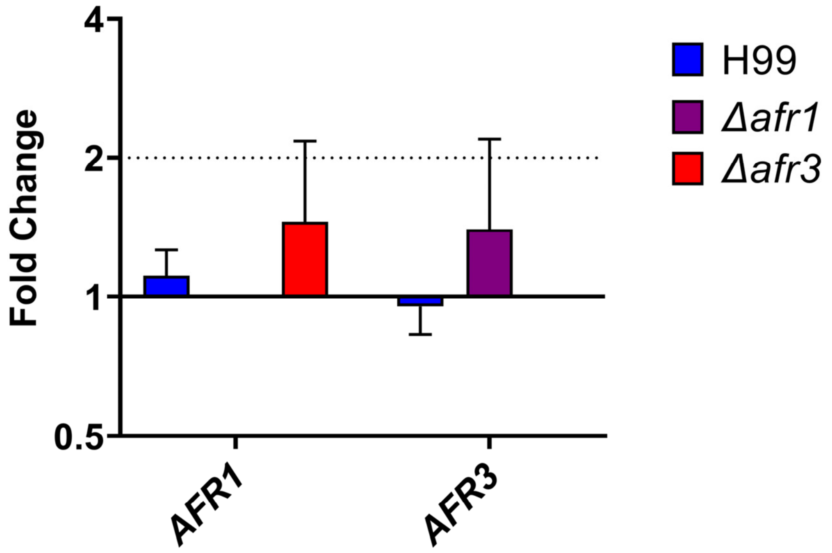

Figure S2. Pump Compensation between Afr1 and Afr3. Cells from H99, *Δafr1*, and *Δafr3* strains had their RNA extracted, followed by cDNA conversion, and expression analysis for *AFR1* and *AFR3*. *ACT1* gene was used as a house keeping control for amplification.

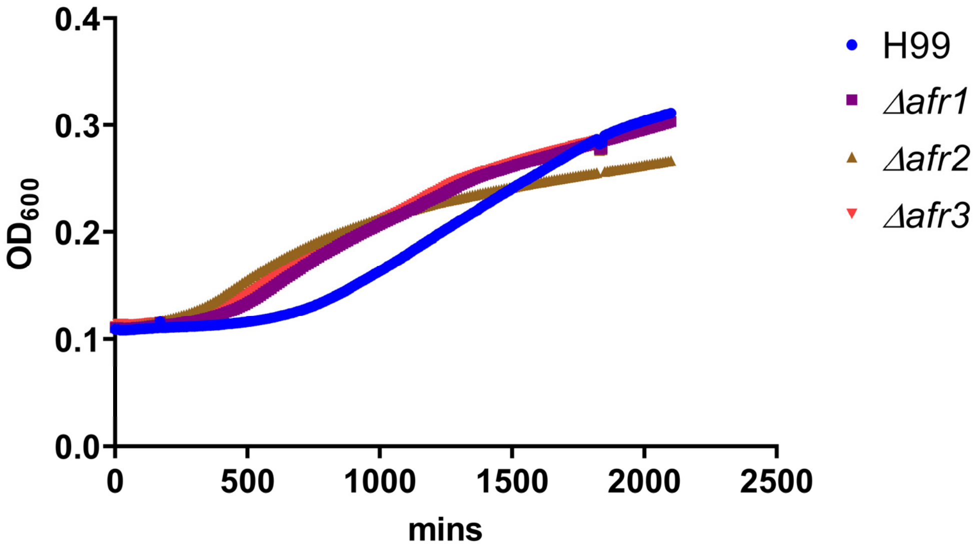

Figure S3. Growth Curves. Cells from H99, *Δafr1*, *Δafr2*, and *Δafr3* strains were monitored in a Spectrophotometer for 72 h, at *37* °C with shaking.
